# Enhanced Salivary and General Oxidative Stress in Hashimoto’s Thyroiditis Women in Euthyreosis

**DOI:** 10.3390/jcm9072102

**Published:** 2020-07-03

**Authors:** Katarzyna Morawska, Mateusz Maciejczyk, Łukasz Popławski, Anna Popławska-Kita, Adam Krętowski, Anna Zalewska

**Affiliations:** 1 Department of Restorative Dentistry, Medical University of Bialystok, 24A M. Sklodowskiej-Curie Street, 15-276 Bialystok, Poland; kmorawska1009@gmail.com; 2 Department of Hygiene, Epidemiology and Ergonomics, Medical University of Bialystok, 2c Mickiewicza Street, 15-022 Bialystok, Poland; 3 Department of Radiology, Medical University of Bialystok, 24A M. Sklodowskiej-Curie Street, 15-276 Bialystok, Poland; gajki91@o2.pl; 4 Department of Endocrinology, Diabetology and Internal Medicine, Medical University of Bialystok, 24A M. Sklodowskiej-Curie Street, 15-276 Bialystok, Poland; annapoplawskakita@op.pl (A.P.-K.); adamkretowski@wp.pl (A.K.); 5 Laboratory of Experimental Dentistry, Medical University of Bialystok, 24A M. Sklodowskiej-Curie Street, 15-276 Bialystok, Poland

**Keywords:** Hashimoto’s disease, oxidative stress, saliva

## Abstract

Hashimoto’s thyroiditis (HT) is one of the most common autoimmune diseases. Although HT is inextricably linked to oxidative stress, there have been no studies assessing salivary redox homeostasis or salivary gland function in patients with HT. This study is the first to compare antioxidant defense and oxidative stress biomarkers in non-stimulated (NWS) and stimulated (SWS) whole saliva and plasma/erythrocytes of HT patients compared to controls. The study included 45 women with HT in the euthyreosis period as well as an age- and gender-matched control group. We showed that NWS secretion was significantly lower in HT patients compared to healthy controls, similar to salivary amylase activity in NWS and SWS. Catalase and peroxidase activities were considerably higher in NWS and SWS of HT patients, while the concentrations of reduced glutathione and uric acid were significantly lower in comparison with healthy subjects. Total antioxidant potential was significantly lower, while total oxidant status and the level of oxidation products of proteins (advanced glycation end products, advanced oxidation protein products) and lipids (malondialdehyde, lipid hydroperoxides) were significantly higher in NWS, SWS and plasma of HT patients. In conclusion, in both salivary glands of women with HT in euthyreosis, the ability to maintain redox homeostasis was hindered. In HT patients we observed oxidative damage to salivary proteins and lipids; thus, some biomarkers of oxidative stress may present a potential diagnostic value.

## 1. Introduction

Hashimoto’s disease (HT) is known as chronic lymphocytic thyroiditis. It is an autoimmune-mediated disease characterized by dense infiltrations of the thyroid gland by plasma cells, macrophages and, particularly, lymphocytes [1,2]. The T and B lymphocytes are stimulated against thyroglobulin and thyroid peroxidase, and induce a number of biochemical processes that lead to progressive destruction of thyrocytes, fibrosis, reduction of the thyroid gland size and its hypofunction [3]. Growing evidence indicates that HT is linked to lowered cellular antioxidant potential and enhanced oxidative stress (OS) [2,3,4].

OS is a situation in which balance between reactive oxygen species (ROS) and the body’s ability to neutralize them is shifted in favor of oxidants [5]. This leads to a temporary or chronic elevation of ROS concentration as well as disturbed cell metabolism and degradation of cell components [5].

Evidence showed an altered antioxidant potential and enhanced OS in the plasma of HT patients. Lassoued et al. [6] demonstrated increased plasma malondialdehyde (MDA) concentration as well as the activities of superoxide dismutase (SOD) and catalase (CAT) compared to the controls. Rostami et al. [2] observed decreased reduced glutathione (GSH) concentration in the plasma of newly diagnosed hypothyroid HT patients. This study showed that GSH depletion initiates OS and development of immunological intolerance in the course of HT. Ates et al. [4] argued that higher OS levels in patients progressing to overt hypothyroidism may be evidence of redox balance shift towards oxidative reactions and could thus serve as a significant factor in the initiation and progression of this disease. Interestingly, in the study performed by Nanda et al. [7], OS levels were higher in the thyroid antibody-positive hypothyroid group than in the thyroid antibody-negative hypothyroid group. The authors concluded that the presence of autoimmune antibodies is a key factor for enhanced ROS production and increased concentrations of oxidative biomolecular modifications, while thyroid hormone levels are of secondary importance.

Systemic inflammation, elevated levels of thyroid antibodies, disturbed concentrations of thyroid hormones and chronically raised ROS levels in the course of HT lead to numerous systemic complications, including cardiological diseases, insulin resistance, mood disorders and salivary gland diseases [2,4,8].

Agha-Hosseini et al. [8] demonstrated significantly reduced unstimulated saliva flow rate and xerostomia among HT women in euthyreosis vs. healthy controls. The cause of salivary gland dysfunction in the course of HT has not been explained yet. It is noteworthy that they observed salivary gland dysfunction in patients in euthyreosis. Abnormalities in both the composition and the amount of the secreted saliva negatively affects oral health and the condition of the entire body. Therefore, it is important to understand the mechanisms leading to salivary gland dysfunction in the course of the described disease. Moreover, the oral cavity is exposed to numerous oxidizing agents capable of generating large amounts of ROS. Evidence showed that salivary and, to some extent, plasma antioxidants, constitute an important part of the antioxidant barrier in both oral cavity and the entire body. Salivary peroxidase (Px), together with catalase (CAT), neutralizes H_2_O_2_ formed in a dismutation reaction catalyzed by superoxide dismutase (SOD). Reduced glutathione (GSH) is the most important low molecular weight salivary antioxidant responsible for maintaining thiol groups of salivary proteins at a reduced level. Forty percent of the total salivary antioxidant barrier is provided by bloodborne uric acid (UA) [5]. Failure of these antioxidant systems may result in the development of oral cavity diseases, including periodontitis [9,10], precancerous lesions [11] and cancers [12]. Previous studies showed the alteration in the salivary antioxidants barrier and the contribution of OS in the development and progression of salivary gland dysfunction in the course of other autoimmune diseases: psoriasis vulgaris, systemic sclerosis, rheumatoid arthritis, diabetes type 1, multiple sclerosis, Sjögren’s syndrome and systemic lupus erythematosus [13,14,15,16,17,18,19,20,21]. In general, reduced/elevated levels of endogenous, non-enzymatic antioxidants or enhanced/weakened activity of antioxidant enzymes and increased oxidative modification of salivary cell components are observed in the saliva of patients with autoimmune diseases. The salivary antioxidants in HT have not yet been determined, so in the light of the above, it appears necessary to assess the antioxidant potential of saliva and the role of OS in the development of salivary gland dysfunction in the course of HT.

The aim of this study was to evaluate antioxidative defense parameters and measurable oxidative stress effects in unstimulated and stimulated saliva and plasma/erythrocytes of patients with HT in euthyreosis and to compare the obtained results with those in the control group.

## 2. Materials and Methods

### 2.1. Patients

The study was approved by the Local Ethics Committee, permission number: R-I-002/386/2016. Each patient was informed of the purpose and the detailed procedure of the study and consented in writing to join the research project.

The study group consisted of 45 women diagnosed with HT. The disease was confirmed when anti-TG and/or anti-TPO levels in serum was above the normal range and occurred with the presence of parenchymal heterogeneity on thyroid ultrasonography (USG). Our experiment included patients with HT in euthyreosis (free thyroxine (fT4) and thyroid- stimulating hormones (TSH) within normal ranges), 24 treated with Eutyrox (doses from 50 to 150 mg; the last tablet taken 24 h before the hormone level test) and 21 untreated. Patients were reported for follow-up visits to the Department of Endocrinology, Diabetology and Internal Medicine of the Medical University of Bialystok. We decided to create only one group, because the results of particular redox balance assays did not differ between patients in the course of a hormone therapy and those not requiring it.

The reference group consisted of 45 generally healthy women, matched to the study group in terms of age and BMI, selected from those who reported for dental check-ups to the Department of Restorative Dentistry, MUB.

Exclusion and inclusion criteria:

Patients with HT and healthy controls did not suffer from any associated diseases, including other autoimmune diseases (type 1 diabetes, rheumatoid arthritis, scleroderma, psoriasis, lupus, Sjögren’s syndrome, etc.) or depression. Participants from both the study and control group were qualified for further examinations only if they did not have periodontitis, gingivitis, or active foci of odontogenic infections. Participants had 18.5 ≤ BMI ≤ 25. The subjects had not taken any drugs that could affect saliva secretion (mainly antidepressants or drugs for hypertension) or its redox status (vitamins, antioxidants) within 3 months prior to saliva collection, nor were they on any reducing diet. Patients and the controls who smoked tobacco or consumed any amount of alcohol or other stimulants were not included in the study. All the subjects in the control group had normal serum TSH, fT4, anti-TG and anti-TPO levels as well as thyroid imaging (homogenous parenchyma without nodules) on USG.

### 2.2. Blood Collection

A total of 10 mL of venous blood samples were collected in ethylenediaminetetraacetic acid (EDTA) tubes. The blood was then centrifuged 1500× *g* at 4 °C for 10 min. The acquired plasma was placed in Eppendorf tubes. The obtained erythrocyte mass was centrifuged three times in cold saline (0.9% NaCl) and then underwent osmotic lysis by adding a cold phosphate buffer (1:9, 50 mM, pH 7.4). In order to prevent sample oxidation and proteolysis, 10 µL 0.5 M BHT (butylated hydroxytoluene, BHT, Sigma-Aldrich, Germany) in acetonitrile was added per 1 mL of plasma and erythrocytes, and stored at −80 °C until assayed, but not for longer than 6 months.

Clinical details of patients and control subjects are presented in Table 1.

### 2.3. Saliva Collection

The assessment of the antioxidant barrier of saliva is complete when it includes analysis of both stimulated and unstimulated saliva. It has been shown that antioxidants produced by the parotid gland are aimed at combating deleterious foreign ROS that may penetrate oral cavity during eating, and antioxidants present in NWS for the rest of the time [22]. The studied material was non-stimulated (NWS) and stimulated (SWS) whole saliva collected via the spitting method. Participants were advised to refrain from consuming meals and drinks other than clean water, performing oral hygiene procedures for 2 h and taking any medications for 8 h before saliva collection. Saliva was collected between 8 a.m. and 10 a.m. to minimize the effect of daily changes on its secretion. The material was taken in a separate room so that patients did not feel uncomfortable or nervous. Participants had their saliva collected in a sitting position, with the head slightly inclined downwards, with minimized face and lip movements, upon a 5-min adaptation period. After that time, every patient rinsed their mouth three times with water at room temperature. The saliva collected during the first minute was discarded. Subsequent batches of saliva (the patient actively spat out the saliva accumulated in the bottom of the oral cavity) were collected into plastic centrifuge tubes placed in ice containers. The time of NWS collection was 15 min [15,16,22]. SWS was collected after a 5-min break, for 5 min. Its stimulation was triggered by dripping 100 µL 2% citric acid under the tongue every 20 s [23]. To avoid sample oxidation, 0.5 M BHT was added to the saliva (Sigma-Aldrich, Saint Louis, MO, USA; 10 µL/mL saliva) [24]. The volume of each sample was measured with a pipette calibrated to 0.1 mL. Saliva secretion was calculated by dividing the volume of the obtained saliva by the number of minutes of its collection. Then saliva was centrifuged (20 min, 4 °C, 10,000× *g*). Further tests were performed using the preserved supernatant fluid, which was frozen at −80 °C until assayed. Frozen samples were stored for no longer than 6 months.

### 2.4. Dental and Periodontal Examination

Dental examination was performed on the day of and immediately after saliva collection using a mirror, an explorer and a periodontal probe, in artificial light, by one calibrated dentist (K.M.). The study included dental evaluation, caries severity index (DMFT) as well as approximal plaque index (API), periodontal probing depth (PPD) and gingival index (GI). DMFT is an index that evaluates the condition of teeth, which consists in counting teeth with caries, removed due to caries or filled because of caries. GI is the assessment of the gingiva for possible inflammation. API is an index used to assess plaque located in interdental spaces. Finally, PPD is an index of the depth of probed gingival pockets. In 20 participants, the study was conducted by another experienced dentist (A. Z.) and the results were compared with those obtained by the head doctor (K. M.). The interrater reliability for DMFT was *r* = 0.92, for GI *r* = 0.94, for API *r* = 0.98 and for PPD *r* = 100.

### 2.5. Xerostomy Assessment and Schirmer Test

The women in both groups were asked to complete a questionnaire containing a list of symptoms associated with xerostomia and xerophthalmia listed in the American-European classification criteria for Sjögren’s syndrome [15,24,25].

Tear secretion was assessed by the Schirmer I test from both eyes for over 5 min with no anesthesia used [26].

### 2.6. Selection of Patients and the Control Group Participants

Blood collection for the determination of hormones, antibodies and other clinical and oxidative stress parameters as well as ultrasound examination took place a day before saliva collection and dental examination, and included 70 patients and 70 control subjects. Based on the results of USG, thyroid hormone and TSH assays, the patients and the control group were qualified for further examinations (over 60 patients (10 patients had elevated TSH levels) and 59 control group participants (11 people from the control group had an abnormal ultrasound examination) were positively qualified for the experiment).

After the dental examination, 15 patients (PPD > 4 mm) and 5 control (gingival bleeding during probing) subjects were eliminated from the experiment due to the coexisting periodontal/gingival inflammation, and 9 additional participants were excluded from the control group as they did not match the other subjects in terms of BMI.

### 2.7. Biochemical Determinations: Salivary Amylase Activity and IL-1β Concentration

The salivary amylase activity (EC 3.2.1.1) was assessed spectrophotometrically using an alkaline solution of 3,5-dinitrosalicylic acid (DNS). The absorbance of samples was measured at 540 nm, accompanying the increased concentration of reducing sugars released during the hydrolysis of starch catalyzed by salivary amylase. Salivary amylase activity was determined in duplicate samples and expressed in μmol/mg protein [23].

Salivary interleukin 1β (IL-1β) concentration was determined by ELISA using a commercially available kit from EIAab Science Inc. Wuhan (Wuhan, China) in accordance with the manufacturer’s instructions provided in the package.

### 2.8. Biochemical Determinations: Redox Assay

All analyses were performed in duplicate samples. Absorbance and fluorescence were measured with Infinite M200 PRO Multimode Tecan microplate reader. The results were standardized to 1 mg of total protein. The content of protein was evaluated by the bicinchoninic method (BCA) using a ready-made reagent kit (Thermo Scientific Pierce BCA Protein Assay Kit, Rockford, IL, USA) and a bovine serum albumin standard (BSA).

#### 2.8.1. Enzymatic Antioxidants

The enzymatic antioxidant barrier was evaluated in saliva and erythrocyte samples by measuring the activity of SOD, CAT, Px and glutathione peroxidase (GPx).

Spectrophotometric evaluation of SOD activity (SOD, E.C. 1.15.1.1) was performed according to Misra and Fridovich [27], based on the adrenaline to adrenochrome oxidation rate. Absorbance measurements were taken at 480 nm wavelength.

Colorimetric measurement of CAT activity (CAT, E.C. 1.11.1.6) was based on the hydrogen peroxide degradation rate [28]. The unit of CAT activity (1 U) was determined as the amount of the enzyme decomposing 1 mmol H_2_O_2_ per minute. The measurements were performed at 240 nm wavelength.

The activity of Px (Px, E.C. 1.11.1.7) was determined colorimetrically according to the method by Mansson-Rahemtulla et al. [29] based on the reduction of 5,5′-dithiobis-(2-nitrobenzoic acid) (DTNB) to thionitrobenzoic acid at 412 nm wavelength.

The activity of GPx (GPx, E.C. 1.11.1.9) was measured spectrophotometrically using the Paglia and Valentine method [30], based on the reduction of organic peroxides in the presence of NADPH at 340 nm.

#### 2.8.2. Non-Enzymatic Antioxidants

The non-enzymatic antioxidant barrier was assessed by measuring GSH and UA concentrations in NWS, SWS and plasma.

GSH concentration was determined colorimetrically based on the reaction with DTNB. Absorbance of the samples was measured at 412 nm wavelength [31].

UA concentration was determined spectrophotometrically at 490 nm using the ability of 2,4,6-Tris(2-pyridyl)-s-triazine to form a blue complex with iron ions in the presence of UA. We used a commercial set of reagents (QuantiChrom^TM^ Uric Acid Assay Kit DIUA-250; BioAssay Systems, Hayward, CA, USA).

Total antioxidant capacity (TAC) level in plasma was determined spectrophotometrically at 660 nm wavelength using 2,2’-azino-bis(3-ethylbenzothiazoline-6-sulphonic acid) (ATBS). The intensity of the color resulting from the reaction of ABTS radical cation was proportional to the content of antioxidants in the tested samples [32].

#### 2.8.3. Total Oxidant Status (TOS) and Oxidative Stress Index (OSI)

TOS and OSI levels were determined in NWS, SWS and plasma.

TOS level was assayed bichromatically (560/800 nm) based on the oxidation of Fe_2+_ to Fe_3+_ in the sample. TOS level was calculated from the standard curve for H^2^O^2^ [33].

OSI was calculated based on the formula OSI = (TOS/TAC)/100 [34].

#### 2.8.4. Oxidative Damage to Proteins and Lipids

Oxidative damage to proteins and lipids was assessed by measuring the concentration of advanced glycation end products (AGE) of proteins, advanced oxidation protein products (AOPP), lipid hydroperoxides (LOOH) and malondialdehyde (MDA) in saliva and plasma samples.

AGE content was assessed by measuring AGE-specific fluorescence at 350/440 nm wavelength, as described by Kalousová et al. [35].

The colorimetric measurement of AOPP content was determined fluorimetrically as described by Kalousová et al. [35] based on the oxidative capacity of iodine ions at 340 nm wavelength.

To measure AGE and AOPP concentrations, NWS, SWS and plasma samples were diluted with phosphate-buffered saline (PBS, pH 7.2) at a ratio of 1:5 (*v*/*v*).

The concentration of LOOH was evaluated spectrophotometrically from the reaction of iron ions (3+) with xylenol orange (XO). The absorbance of Fe-XO complex was measured at 560 nm wavelength [36].

MDA concentration was assessed colorimetrically based on the reaction with thiobarbituric acid (TBA) at 535 nm wavelength. 1,1,3,3-Tetraethoxypropane was used as a standard [24].

### 2.9. Statistical Analysis

Statistical analysis was performed using GraphPad Prism 8.3.0 for MacOS (GraphPad Software, Inc. La Jolla, CA, USA). The distribution of the obtained results was assessed using the Shapiro–Wilk test. Due to the lack of normal distribution, the Mann–Whitney U test was used for quantitative comparisons. Chi-square test with Yates’s modification was used to analyze the differences in the prevalence of qualitative variables. Correlations of the results were assessed using the Spearman rank correlation coefficient. The statistical significance level was set at *p* < 0.05.

The number of subjects was determined based on our previous experiment, assuming that the power of the test would be equal to 0.9 (ClinCalc sample size calculator).

## 3. Results

### 3.1. Clinical Data

Saliva secretion in the group of HT patients was 32% lower than in the control group (*p* = 0.02). None of the patients had the rate of unstimulated saliva secretion below 0.1 mL/min. Moreover, stimulated saliva secretion did not differ between the groups. The concentration of total protein in unstimulated and stimulated saliva of HT patients was significantly higher than in the control group (↑46%, *p* = 0.00002 and ↑16%, *p* = 0.0003, respectively). Salivary amylase activity was considerably lower in NWS (↓50%, *p* = 0.00001) and SWS (↓114%, *p* = 0.00001) of HT patients compared to the controls (Table 2).

IL-1β concentration in NWS and SWS of patients with HT was significantly higher compared to the controls (↑119%, *p* = 0.00001 and, ↑52%, *p* = 0.00002, respectively).

Oral dryness was more prevalent in HT women than in healthy controls (*p* = 0.003). The prevalence of eye dryness was similar in both examined groups.

The median value of Schirmer I test for the left and right eye did not differ between the groups.

The analysis of dental data showed no differences in DMFT, API, GI and PPD between the study and the control group (Table 2).

### 3.2. Antioxidant Defense Parameters

#### 3.2.1. NWS

**Figure 1:**
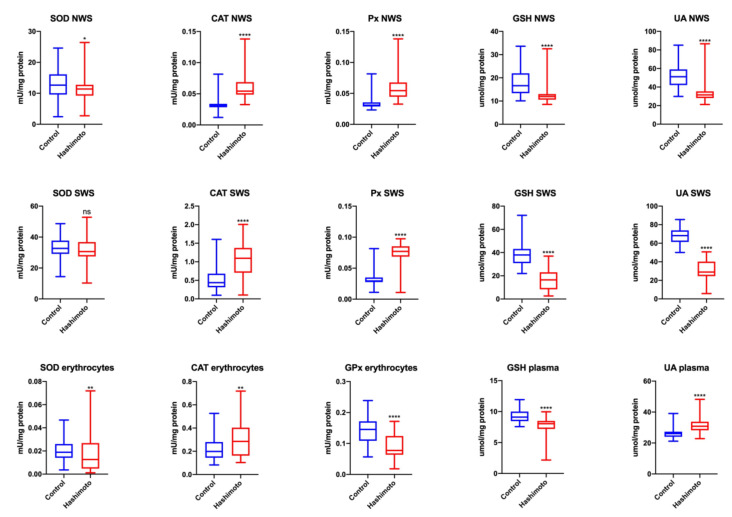
Enzymatic and nonenzymatic antioxidants in NWS, SWS and blood plasma/erythrocytes of the patients and control group. Data are shown as median (minimum–maximum). CAT—catalase; GPx—glutathione peroxidase; GSH—reduced glutathione; NWS—non-stimulated whole saliva; Px—salivary peroxidase; SOD—superoxide dismutase-1; SWS—stimulated whole saliva; UA—uric acid. * *p* < 0.05, ** *p* < 0.01, and **** *p* < 0.0001.

SOD activity as well as the levels of GSH, UA and TAC (Figure 1) in NWS of HT patients were significantly lower than those in NWS of control patients (↓10%, *p* = 0.03; ↓28%, *p* = 0.00003; ↓38%, *p* = 0.00003; ↓34%, *p* = 0.00005, respectively). CAT and Px activities in NWS of HT patients were notably higher compared to those parameters found in NWS of the control group (↑66%, *p* = 0.00002; ↑66%, *p* = 0.00003, respectively) (Figure 1).

#### 3.2.2. SWS

**Figure 2:**
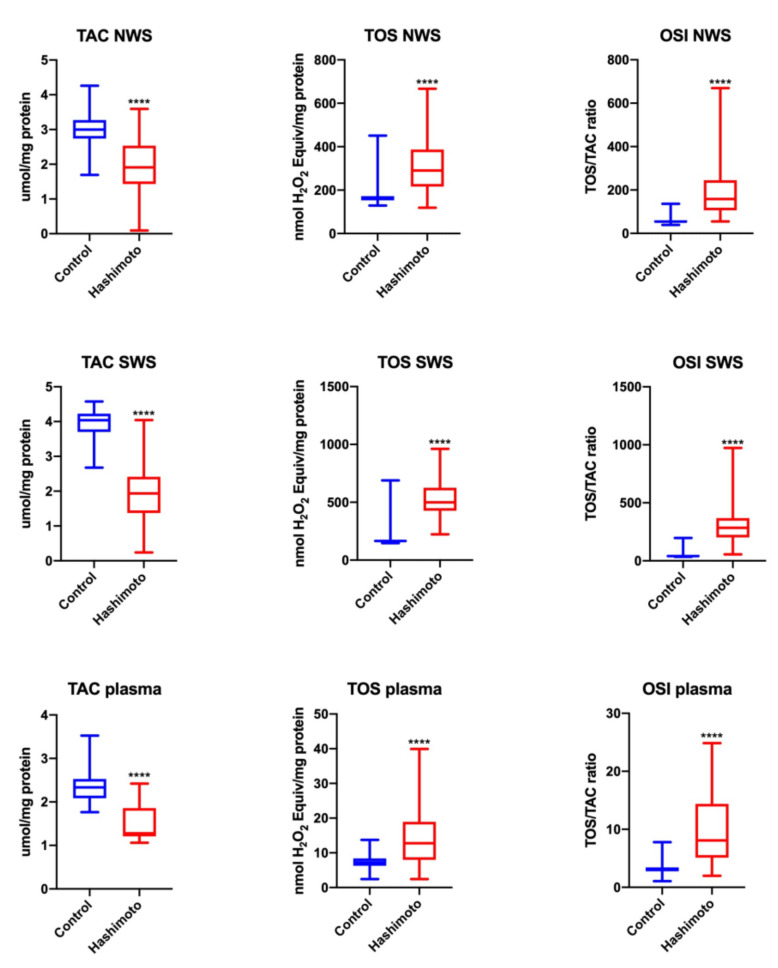
Redox status in NWS, SWS and plasma of the patients and control group. Data are shown as median (minimum–maximum). OSI—oxidative stress index; NWS—non-stimulated whole saliva; SWS—stimulated whole saliva; TAC—total antioxidant capacity; TOS— total oxidant status. **** *p* < 0.0001.

GSH, UA and TAC (Figure 2) concentrations in NWS of HT patients were significantly lower than in NWS of healthy controls (↓57%, *p* = 0.000003; ↓58%, *p* = 0.00001; ↓53%, *p* = 0.00001, respectively). CAT and Px activities in NWS of HT patients were considerably higher than in NWS of the control group (↑147%, *p* = 0.00002; ↑166%, *p* = 0.00003, respectively) (Figure 1).

#### 3.2.3. Erythrocytes, Plasma

SOD and GPx activities in blood erythrocytes as well as GSH and TAC plasma concentrations (Figure 2) in HT patients were significantly lower than the discussed parameters in the erythrocytes and plasma of the control group (↓50%, *p* = 0.009; ↓45%, *p* = 0.00001; ↓13%, *p* = 0.001; ↓82%, *p* = 0.00001, respectively) (Figure 1).

#### 3.2.4. TOS and OSI

We observed significantly increased values of TOS and OSI in NWS (↑81%, *p* = 0.00001; ↑191%, *p* = 0.000001, respectively), SWS (↑201%, *p* = 0.00001; ↑588%, *p* = 0.000001, respectively) and plasma (↑76%, *p* = 0.00001; ↑158%, *p* = 0.000001, respectively) of HT patients compared to the control group (Figure 2).

#### 3.2.5. Products of Oxidative Modifications

**Figure 3:**
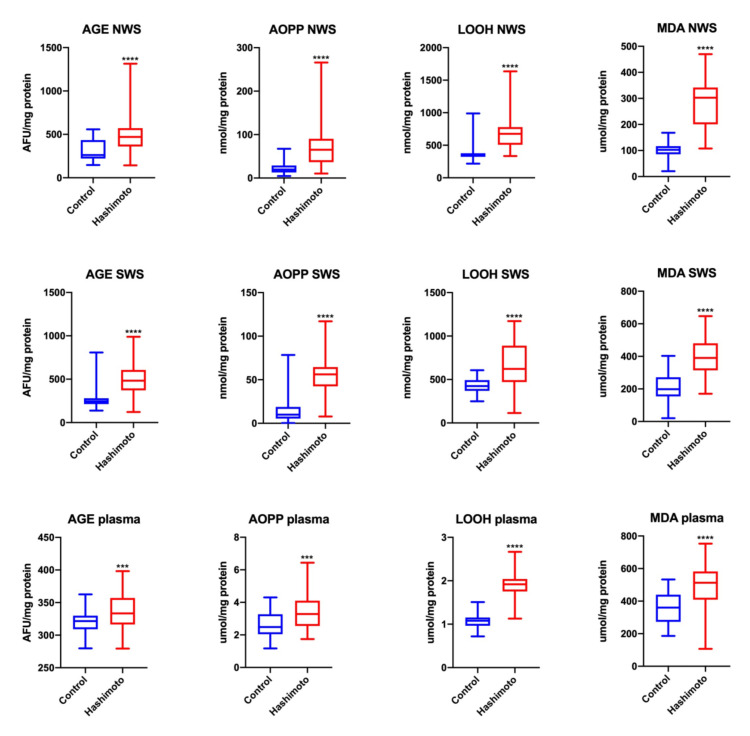
Oxidative damage in NWS, SWS and plasma of the patients and control group. Data are shown as median (minimum–maximum). AGE—advanced glycation end products; AOPP—advanced oxidation protein products; LOOH—lipid hydroperoxides; MDA—malondialdehyde; NWS—non-stimulated whole saliva; SWS—stimulated whole saliva. *** *p* < 0.001, and **** *p* < 0.0001.

The concentrations of all the evaluated products of oxidative modifications: AGE, AOPP, LOOH and MDA were considerably higher in NWS (↑80%, *p* = 0.00001; ↑232%, *p* = 0.00002; ↑91%, *p* = 0.00001; ↑194%, *p* = 0.00001, respectively), SWS (↑97%, *p* = 0.00001; ↑476%, *p* = 0.00001; ↑46%, *p* = 0.00001; ↑96%, *p* = 0.00001, respectively) and plasma (↑3%, *p* = 0.0007; ↑31%, *p* = 0.0003; ↑77%, *p* = 0.00001; ↑42%, *p* = 0.00001, respectively) of HT patients compared to the values of these parameters obtained in the control group (Figure 3).

#### 3.2.6. Comparison of Antioxidants and Redox Balance Markers between NWS and SWS

##### Control

We observed significantly higher activity of SOD, CAT, Px (*p* < 0.0001, *p* < 0.0001, *p* < 0.0001, respectively) and GSH, UA, TAC, OSI, AOPP, LOOH and MDA (*p* < 0.0001, *p* < 0.0001, *p* < 0.0001, p < 0.0001, *p* < 0.0001, *p* < 0.0001, *p* < 0.0001, respectively) concentration in SWS vs. NWS of the control women.

##### HT Women

The activity of SOD, CAT, Px (*p* < 0.0001, *p* < 0.0001, *p* < 0.0001, respectively) and GSH, TOS, OSI and MDA (*p* = 0.025, *p* < 0.0001, *p* < 0.0001, *p* < 0.0001, respectively) concentrations were significantly higher in SWS compared to NWS of HT women (Table 3).

#### 3.2.7. Saliva–Blood Ratio

##### NWS

CAT, Px, AGE AOPP, MDA (*p* = 0.01, *p* < 0.0001, *p* < 0.0001, *p* < 0.0001, *p* < 0.0001, respectively) salivary/blood ratio was significantly higher in NWS of HT women vs. control, while GSH and UA (*p* = 0.0009, *p* < 0.0001) salivary/blood ratio was significantly lower in NWS of HT women vs. control.

##### SWS

CAT, Px, TOS, OSI, AGE, AOPP and MDA (*p* = 0.005, *p* < 0.0001, *p* < 0.0001, *p* < 0.0001, *p* < 0.0001, *p* < 0.0001, *p* < 0.0001, *p* = 0.002, respectively) salivary/blood ratio was significantly higher in SWS of HT women vs. control, while GSH, UA, TAC and LOOH (*p* < 0.0001, *p* < 0.0001, *p* < 0.0001, *p* = 0.01, respectively) salivary/blood ratio was significantly lower in SWS of HT women vs. control (Table 4).

#### 3.2.8. Correlations

In the study group we demonstrated a negative correlation between the plasma concentrations of anti-TPO antibodies and GSH (*r* = −0.854, *p* < 0.0001). We also observed a negative correlation between TAC in NWS (*r* = −0.704, *p* < 0.0001) and SWS (*r* = −0.759, *p* < 0.0001) and serum concentration of anti-TPO.

We also found a positive correlation between MDA in NWS and thyroglobulin antibodies (r = 0.851, *p* < 0.0001) as well as between LOOH in SWS and anti-TG (r = 0.839, *p* < 0.0001).

We showed a negative correlation between SOD activity in SWS and plasma glucose concentration (*r* = −0.851, *p* < 0.0001) in the HT group.

In the study group we found a negative correlation between GSH and AOPP concentrations in SWS (*r* = −0.730, *p* < 0.0001). Moreover, IL-1β correlated positively with LOOH concentrations (r = 0.886, *p* < 0.0001) and negatively with GSH levels in SWS (*r* = −0.849, *p* < 0.0001).

The minute flow of unstimulated saliva correlated negatively with IL-1β concentration in NWS (*r* = −0.891, *p* < 0.0001). We also observed a negative correlation between salivary α-amylase activity and TOS in NWS (*r* = −0.8, *p* < 0.0001). The remaining correlations were included in the supplementary materials.

## 4. Discussion

In the presented experiment we employed a wide range of biochemical assays to search for a link between OS expressed as antioxidant activity/concentration and oxidative damage products and salivary gland function in patients with HT in euthyreosis. To the best of our knowledge, this study is the first to assess the function of salivary glands and their redox balance in patients with HT in euthyreosis.

OS is the result of an imbalance between ROS production and neutralization, which leads to oxidative damage to tissues. In the case of autoimmune thyroiditis, OS is considered to be the result of a deficiency of thyroid hormones as well as autoimmunity and the associated inflammation. During the synthesis of thyroid hormones, iodine is oxidized by nicotinamide adenine dinucleotide phosphate oxidase (NOX). Hydrogen peroxide (H_2_O_2_) formed in this reaction is used for the production of thyroid hormones [37]. Excess of H_2_O_2_, e.g., due to excessive iodine substitution or deficiency of glutathione peroxidase (GPx, an enzyme involved in the neutralization of hydrogen peroxide and protecting thyroid tissue from oxidative damage), leads to apoptosis and necrosis of thyrocytes. Interestingly, in the presented study we demonstrated a 45% decrease of GPx activity in blood erythrocytes of HT patients, which, in our opinion, largely contributes to the observed increase in the generation of oxygen free radicals (↑76% TOS in serum). In the situation of increased concentration of ROS, particularly H_2_O_2_, increased immunogenicity of thyroid specific antigens (thyroglobulin and thyroid peroxidase) and intensified intercellular adhesion molecule-1 (ICAM-1) expression are observed, which results in increased generation of antibodies and autoimmune response as well as raises in inflammation [38]. The latter, in turn, directly increases H_2_O_2_ in thyroid epithelial cells and activates NOX in T and B lymphocytes, which further boosts ROS production [3].

The existence and extent of OS can be assessed based on the behavior of numerous biomarkers, including the measurement of the concentration/activity of antioxidants as well as the evaluation of the concentrations of oxidative modification products [39]. In our research, we used a wide panel of biomarkers of oxidative stress, because there is a belief that a single parameter does not reflect the size and severity of the OS phenomenon [40]. The parameters helpful in the assessment of OS are also total antioxidant capacity (TAC), total oxidative status (TOS) and oxidative stress index (OSI) [41]. It has been evidenced that TAC expresses the efficiency of both enzymatic and non-enzymatic antioxidant defense mechanisms, whereas TOS is the sum of all oxidants present in the sample. OSI illustrates the relationship between antioxidant mechanisms and oxidative molecules [42].

Our results showed a decrease in antioxidant potential (82%↓TAC) and increased production of free radicals as well as all products of oxidative modifications in the serum of patients with HT in euthyreosis compared to the controls, which confirms a shift in the redox balance towards oxidation and the existence of general OS. Decreased concentration of GSH deserves special attention as the reduction of GSH concentration is considered a causative factor in the development of autoimmune diseases by inhibition of IL-1 and T-cell receptor-mediated transduction signaling [1]. Increased concentration of GSH has been evidenced to be the result of GSH use through oxidation, conjugation or extrusion from the cell, and indicates highly raised OS [40]. Interestingly, we demonstrated a negative correlation between anti-TPO antibody and GSH concentrations, which is consistent with the results of Rostami et al. [2]. According to these authors, their findings prove that the presence of antibodies is a causative factor for excessive ROS production and that GSH is capable of inhibiting complement-mediated damage in HT. They also presume that GSH deficiency may indicate the occurrence of processes leading to oxidative stress activation and the development of immune intolerance.

Interestingly, changes in the salivary redox balance seem to reflect those observed in the blood, but it should be noted that the changes in saliva are more intense. Only for UA we see different directionality. Its reduced salivary concentration in patients with HT vs. control suggests that in the oral cavity UA behaves as an antioxidant. It has been shown, that the increased concentration of UA, that we observe in the serum of patients with HT, shifts the redox balance towards the oxidation reaction and OS and may be a factor predisposing to cardiovascular diseases [43]. Moreover, the performed analyses did not reveal any correlation between the redox balance parameters in blood and saliva. The lack of correlation and saliva/blood ratio analysis may suggest that oxidative stress in the salivary glands is independent of general oxidative stress in the course of HT. What’s more, the higher saliva/blood ratio with respect to some of the parameters studied, in the HT women group compared to the control, indicates that the observed changes are the result of processes occurring in the salivary glands, and are not the result of their passive blood transitions.

The large salivary glands together produce about 90% of the total saliva volume. The largest of them—the parotid gland—produces saliva mainly in response to the applied stimulation, hence any changes in stimulated saliva composition and amount are considered to reflect the function of the parotid gland. At rest, 2/3 of the total saliva amount is produced by the submandibular glands. Therefore, any variations in the composition and amount of NWS reflect the functioning of the latter glands [44,45].

Our study demonstrated a reduction in the antioxidant potential of parotid and submandibular salivary glands, confirmed by a 34% decrease in TAC in NWS and a 53% decrease in TAC in SWS, which manifests the inefficiency of antioxidant systems of these glands to eradicate ROS. Although we observed significantly increased CAT and Px activities in NWS and SWS, which may—to some extent—prove an adaptive mechanism of the salivary glands in response to excessive ROS production and be of great importance to oral health. Px and CAT maintain concentration salivary H_2_O_2_ at a level 8 to 14 µM [46], which is non-toxic to oral fibroblasts [47] and epithelial cells [48].

In the euthyreosis status in the course of HT, the causes of reduced antioxidant response should be seen as a result of depletion of the resources in the process of ROS neutralization or of the oxidative modification of polypeptide chains rather than as a result of decreased synthesis caused by reduced production of thyroid hormones. The impaired antioxidant defense may also be caused by non-enzymatic glycation of these enzymes, which explains the negative correlation between SOD activity in SWS and plasma glucose concentration in HT group [49], despite the fact that diabetes as well as insulin resistance were excluded in HT patients. Moreover, the negative correlation between TAC in NWS and SWS and the serum concentration of anti-TPO as well as between GSH and IL-1β concentrations in SWS confirm that the exhaustion of antioxidant sources in salivary glands is related to an elevated oxidative stress level due to autoimmunity-related inflammation and that increased concentration of autoimmune antibodies is a key factor for the enhancement of ROS production.

As our results show, the impaired saliva antioxidant barrier results in an increase in oxidative modifications of salivary proteins and lipids. So it is advisable to use exogenous antioxidants supporting endogenous antioxidant mechanisms. The results of our study revealed a significantly greater percentage increase in the concentration of TOS and OSI in SWS (↑201% and ↑588%, respectively) compared to NWS (↑81% and ↑191%, respectively) in HT female patients. A significant reduction of GSH concentration in SWS (↓57%) vs. NWS (↓23%) in HT women as well as a negative correlation between AOPP and GSH levels in SWS may be helpful in understanding the intensification of oxidative modifications to proteins in the parotid vs. submandibular salivary glands. It has been evidenced that the main role of GSH is in maintaining thiol groups of proteins at a reduced level, i.e., protecting proteins against oxidation [50,51]. We also found a positive correlation between MDA in NWS and anti-TG, LOOH in SWS as well as between anti-TG and LOOH in SWS and IL-1β concentration in SWS, which proves that oxidative damage to the lipids contained in salivary glands is boosted with an increase in autoimmunity-related inflammation in the course of HT.

The main physiological difference between the parotid and submandibular saliva is the fact that the former type of saliva is secreted mainly during eating, whereas the latter type is produced continuously and is responsible for maintaining the integrity of oral structures [22]. Maintaining the appropriate rate of both types of secretion is therefore equally important for the functioning of the body. Despite a higher intensity of OS and a more pronounced decrease in antioxidant defense in the parotid glands, the rate of stimulated saliva secretion did not differ significantly between the groups. However, our study demonstrated that HT patients lose the unstimulated salivary gland function. The median values of NWS and SWS secretion were within the standard limits assumed for proper saliva secretion. It is noteworthy that in 15 patients, NWS secretion was lower than 0.2 mL/min (but higher than 0.1 mL/min) and 5 HT group patients secreted stimulated saliva at a level lower than 0.7 mL/min, which proves the developed salivary gland failure in these patients, referred to as hyposalivation, and explains the significant intensification of subjective symptoms of reduced saliva secretion reported when completing the survey on a dry mouth. The performed analyses excluded Sjögren’s syndrome, but, to be 100% certain, a biopsy of the salivary glands would be advisable. However, we were not granted permission for its performance from the Bioethics Committee. Evidence showed that proinflammatory cytokines, including the evaluated IL-1β, induce the activity of metalloproteinases, which leads to changes in the basement membrane of the salivary glands and the structure of receptors for neurotransmitters associated with saliva secretion [52]. A higher increase in IL-1β concentration in NWS (↑119%) vs. SWS (↑52%) as well as in the observed relationship between NWS and IL-1β concentration may confirm the destruction of acinar cell-basement membrane interaction by excessive production of MMPs followed by a decreased number of secretory units (acini and ducts) [53,54]. It has also been demonstrated that the presence of IL-β in the inflamed environment may inhibit the release of acetylcholine from the residual nerves, resulting in reduced saliva secretion [13,14,55]. The described phenomenon of extracellular matrix remodeling has been demonstrated as a cause of reduced saliva secretion in Sjögren’s syndrome patients [56] and reduced activity of muscarinic neurotransmitters/receptors in the submandibular glands of diabetic patients [55].

Changes in salivary amylase activity are considered a determinant of sympathetic nervous system (SNS) activity. The stimulation of β-adrenergic receptor activity results in increased production/secretion and activity of salivary α-amylase and other salivary proteins [57]. In our study, we observed decreased salivary α-amylase activity with a simultaneous increase in protein concentration in both SWS and NWS, which could suggest a higher activity of this branch of autonomic nervous system. The negative correlation between salivary α-amylase activity and TOS in NWS may suggest that lowered activity of salivary α-amylase results from the use of this enzyme in the elimination of excessive amounts of ROS (↑TOS) or, more likely, from the oxidative modification of its polypeptide chain, leading to a loss of/significant reduction in enzymatic activity.

One of the limitations of this paper is the fact of determining only some parameters characterizing the redox balance. Perhaps the use of other biomarkers would change the results and conclusions, which of course can be considered a weak point of this experiment.

## 5. Conclusions

(1) Parotid as well as submandibular salivary glands of HT female patients in euthyreosis had an impaired ability to maintain the redox status at the level observed in the salivary glands of the control women.
(2) The saliva of patients with HT in euthyreosis demonstrated a reduced antioxidant potential. Moreover, a significant increase in oxidatively modified molecules in NWS and SWS suggests the failure of the salivary gland antioxidant barrier to combat excess ROS production.
(3) OS in NWS and SWS of HT women appears to be closely connected with autoimmunity-related inflammation, and not with the level of thyroid hormones or TSH.
(4) The secretory function of the submandibular glands of HT female patients in euthyreosis is decreased, which is manifested as a significant reduction of unstimulated saliva secretion.

## Figures and Tables

**Table 1:** Clinical characteristics of the patients and control group.

Patients Variables	Control, *n* = 45 M (Min–Max)	HT, *n* = 45 M (Min–Max)	*p*
Age (years)	35 (29–43)	35 (29–43)	NS
BMI (kg/m^2^)	19.25 (18.3–24.52)	23.15 (18.19–25.89)	NS
TSH (µU/mL)	1,99 (0.35–2.85)	2.85 (0.35–4.94)	NS
Free T4 (ng/mL)	1.2 (0.91–1.4)	1.1 (0.7–1.42)	NS
Free T3 (pg/mL)	2.56 (1.9–3.45)	2.4 (1.7–3.65)	NS
Anty TPO (IU/mL)	0.35 (0–2.1)	321.2 (108.25–652.1)	<0.0001
Anty TG (IU/mL)	0.32 (0–2.1)	153.8 (99.9–333.1)	<0.0001
PTH (pg/mL)	39.45 (10–62.2)	35.02 (15.51–63.56)	NS
Glucose (mg/dL)	86.37 (76.96–95.49)	84.11 (73.90–98.65)	NS
Euthyrox, n(%)	0	24 (53.3%)	

Abbreviations: HT—Hashimoto thyroiditis, C—control, BMI—body mass index, TSH—thyrotropic hormone, anty TPO—thyroid peroxidase antibody, anty TG—thyroid peroxidase antibody, PTH—parathyroid hormone.

**Table 2:** Salivary gland function and dental indices of the patients and the control group.

Clinical Parameters	Control			HT			*p*
	Median	Minimum	Maximum	Median	Minimum	Maximum	
NWS mL/min	0.51	0.27	0.96	0.32	0.07	0.77	0.02
SWS mL/min	1.01	0.9	2	0.9	0.2	2	ns
TP NWS (mg/mL)	704.2	556.5	924.5	1293	979.4	1707	<0.0001
TP SWS (mg/mL)	1545	371.3	1672	1834	266.6	3861	<0.0001
Salivary amylase NWS (μmol/mg protein)	0.22	0.18	0.28	0.11	0.09	0.15	<0.0001
Salivary amylase SWS (μmol/mg protein)	0.3	0.19	0.38	0.14	0.1	0.16	<0.0001
Salivary IL-1β NWS (pg/mg protein)	0.91	0.27	2	2	0.4	3	<0.0001
Salivary IL-1β SWS (pg/mg)	5.1	2.7	6.8	7.8	4	12	<0.0001
Schirmer- I test (mm/5 min) Left eye Right eye	21 25	10 11	28 30	19 20	12 9	23 30	ns
Subjective dryness n (%) Xerostomiaxerophtalmia	1(2.22) 3(6.67)			26(57.7) 3(6.67)			0.003 ns
DMFT	15	0	25	16	3	28	ns
API	41.85	0	100	54.5	8.3	100	ns
GI	1	0	2	1	0	2	ns
PPD (mm)	1.898	1.14	2.55	1.99	1.33	3.64	ns

NWS—unstimulated saliva, SWS—stimulated saliva, TP—total protein, DMFT—decay, missing, filling teeth, API—approximal plaque index, GI—gingival index, PPD—periodontal pocket depth, ns—non-statistically important.

**Table 3:** Comparison of NWS to SWS.

Redox Parameters	Control							HT						
	NWS			SWS			*p*	NWS			SWS			*p*
	Median	Minimum	Maximum	Median	Minimum	Maximum		Median	Minimum	Maximum	Median	Minimum	Maximum	
SOD	12.63	2.439	24.6	32.67	14.42	48.69	<0.0001	11.38	2.746	26.41	30.61	10.28	52.8	<0.0001
CAT	0.0306	0.0121	0.0815	0.4379	0.1	1.603	<0.0001	0.0544	0.0326	0.1381	1.094	0.1058	2.006	<0.0001
Px	0.031	0.0234	0.0815	0.4379	0.1	1.603	<0.0001	0.0545	0.0326	0.1381	1.094	0.1058	2.006	<0.0001
GSH	16.61	10.09	33.62	37.94	21.98	72.11	<0.0001	11.94	8.543	32.52	16.52	2.634	36.96	0.0258
UA	51.12	29.86	85.09	68.21	50.05	85.52	<0.0001	31.53	21.19	86.65	28.98	5.787	50.67	ns
TAC	2.997	1.695	4.258	4.038	2.677	4.578	<0.0001	1.909	0.0935	3.594	1.934	0.2413	4.044	ns
TOS	160.3	129.1	451.2	165.4	147.2	689.9	ns	290.1	118.7	666.8	499.2	223.6	961.4	<0.0001
OSI	54.48	39.2	136.5	41.24	34.02	196.7	<0.0001	158.5	54.79	669.8	284	55.28	973.4	<0.0001
AGE	261.6	146.4	558.1	244.5	138.5	807.9	ns	471	143.2	1314	482.9	122	989.2	ns
AOPP	19.55	4.569	67.25	9.754	0.1632	78.37	<0.0001	65.01	10.37	265.8	56.21	7.779	117	ns
LOOH	352	218.1	989.5	424.5	249.6	607.5	<0.0001	675.2	334.4	1635	622.2	114.3	1172	ns
MDA	102.6	20.49	168.2	198.4	20.46	402.8	<0.0001	302.6	107.6	469.9	390.1	170.2	647.3	<0.0001

AGE—advanced glycation end products; AOPP—advanced oxidation protein products; AUC—area under the curve; CAT—catalase; GPx—glutathione peroxidase; GSH—reduced glutathione; LOOH—lipid hydroperoxides; MDA—malondialdehyde; NWS—non-stimulated whole saliva; OSI—oxidative stress index; Px—salivary peroxidase; ROS—reactive oxygen species; SOD—superoxide dismutase; SWS—stimulated whole saliva; TAC—total antioxidant capacity; TOS—total oxidant status; UA—uric acid.

**Table 4:** Saliva to blood ratio.

Salivary/Blood Ratio	Control			HT			*p*
	Median	Minimum	Maximum	Median	Minimum	Maximum	
SOD NWS	655.6	145.7	3580	816.4	113.1	10,113	ns
SOD SWS	1625	396.1	10,721	2312	414.4	29,459	ns
CAT NWS	0.1582	0.0411	0.8706	0.2144	0.0501	0.8531	0.0115
CAT SWS	1.988	0.3915	10.61	3.337	0.3794	14.38	0.0053
Px NWS	0.2378	0.1272	0.9917	0.6863	0.2076	4.777	<0.0001
Px SWS	3.186	0.6492	18.57	11.55	1.403	71.15	<0.0001
GSH NWS	1.819	0.9336	3.613	1.464	0.9591	8.705	0.0009
GSH SWS	4.051	2.369	9.534	2.216	0.5282	3.961	<0.0001
UA NWS	1.973	1.002	2.763	1.034	0.6256	2.508	<0.0001
UA SWS	2.525	1.851	3.556	0.9855	0.2191	1.775	<0.0001
TAC NWS	1.246	0.8213	2.401	1.356	0.0647	2.665	ns
TAC SWS	1.728	0.9485	2.466	1.268	0.1385	2.794	<0.0001
TOS NWS	22.5	11.08	54.29	23.78	6.919	161.5	ns
TOS SWS	23.61	11.63	100.9	38.81	9.852	155.5	<0.0001
OSI NWS	18.33	7.485	43.07	15.68	3.368	184	ns
OSI SWS	13.77	7.244	54	30.15	6.401	429.8	<0.0001
AGE NWS	0.8406	0.4666	1.786	1.366	0.4045	4.279	<0.0001
AGE SWS	0.761	0.4457	2.84	1.434	0.3307	3.133	<0.0001
AOPP NWS	7.951	1.338	31	18.88	2.203	69.16	<0.0001
AOPP SWS	3.725	0.0983	29.82	16.57	1.595	36.84	<0.0001
LOOH NWS	327.5	214.4	1255	334.5	177.6	805.8	ns
LOOH SWS	419.4	249.7	677	341.4	65.17	637	0.0135
MDA NWS	0.2532	0.0631	0.6355	0.5392	0.2124	3.402	<0.0001
MDA SWS	0.5698	0.0548	1.38	0.8321	0.2707	3.756	0.0024

AGE—advanced glycation end products; AOPP—advanced oxidation protein products; AUC—area under the curve; CAT—catalase; GPx—glutathione peroxidase; GSH—reduced glutathione; LOOH—lipid hydroperoxides; MDA—malondialdehyde; NWS—non-stimulated whole saliva; OSI—oxidative stress index; Px—salivary peroxidase; ROS—reactive oxygen species; SOD—superoxide dismutase; SWS—stimulated whole saliva; TAC—total antioxidant capacity; TOS—total oxidant status; UA—uric acid.

## Supplementary Materials

The following are available online at https://www.mdpi.com/2077-0383/9/7/2102/s1, Table S1: Spearman *r* coeficient of variables, Table S2: *p* value of correlations.
